# Gambling disorder and other behavioral addictions: mechanisms, recognition and treatment

**DOI:** 10.1192/j.eurpsy.2023.28

**Published:** 2023-07-19

**Authors:** A. Goudriaan

**Affiliations:** ^1^Psychiatry, Amsterdam University Medical Centers; ^2^Amsterdam Institute for Addiction Research, Jellinek, Arkin, Amsterdam, Netherlands

## Abstract

**Abstract:**

Gambling disorder was reclassified as a first behavioral addiction in the DSM-5 in 2013. In 2018, gaming disorder was included in the ICD-11. In this presentation, research findings regarding the psychological and neurobiological mechanisms in gambling disorder and other behavioral addictions are highlighted. Specifically, neural and behavioral responses regarding reward expectation, reward outcomes, and the role of specific mechanisms in gambling like near wins, and in gaming, like lootboxes are highlighted. Besides the recognition of gambling disorder, the broader perspective of gambling harms is highlighted and related to psychosocial, financial, and health consequences. The perspective of interaction between human design factors (e.g. psychological and neurobiological mechanisms, such as decision making, reward processing) and gambling design factors (e.g. near wins, multiline betting) is reviewed. Evidence with regard to psychological and pharmacological interventions are discussed and future directions for research perspectives are discussed.

**Disclosure of Interest:**

A. Goudriaan Grant / Research support from: Anna Goudriaan received grants from the Dutch Foundation for Scientific Research (NWO and ZonMw) and from VCVGZ Foundation

